# The effect of tongue base suspension with uvulopalato-pharyngoplasty on sleep quality in obstructive sleep apnea

**DOI:** 10.1038/s41598-018-27094-w

**Published:** 2018-06-08

**Authors:** Yung-An Tsou, Chen-Wei Huang, Tsu-Fang Wu, Lung-Wen Hung, Wen-Dien Chang

**Affiliations:** 1 Department of Otolaryngology-Head and Neck Surgery, China Medical University Hospital, Taichung, Taiwan; 2 Department of otolaryngology Head and Neck surgery, China Medical University Hospital, Taichung, Taiwan; 3 Graduate Institute of Biomedicine Sciences, China Medical University, Taichung, Taiwan; 4 Depratment of Sleep Medicine, China Medical University Hospital, Taichung, Taiwan; 5 Department of Sports Medicine, China Medical University, Taichung, Taiwan

## Abstract

The objective was to investigate whether tongue base suspension with uvulopalatopharyngoplasty (UPPP) is beneficial on polysomnography analysis for sleep quality in patients with obstructive sleep apnea (OSA) anatomically classified as Fujita type III (small tonsils and a bulky tongue base). In the retrospective study, the charts of 36 patients with OSA that underwent tongue base suspension with UPPP from 2012 through 2015 were reviewed. The surgical outcome measured according to Sher’s classification (AHI reduction > 50% and AHI < 20 per hour as success group, otherwise as failure group). The pre- and post-operative sleep quality parameters were evaluated, and the total sleep time changes were evaluated based on electroencephalography study, slow wave sleep, sleep efficiency, rapid eye movement sleep percentile, and Epworth sleep scale scores. Respiratory, the outcomes of polysomnography analysis were then compared between the successful surgery and surgical failure groups during a 1-year follow up. Total arousals and reduced respiratory arousal indices, along with unchanged periodic leg movement and spontaneous electroencephalography arousal indices, were observed in the successful surgery group but not in the surgical failure group. There were 66% resulted in surgical success by this surgery, and 34% as in failure group according to Sher’s criteria. Patient sleep quality was further improved by reducing the respiratory arousal index and increasing the rapid eye movement sleep percentile during the 1-year follow up.

## Introduction

The prevalence of obstructive sleep apnea (OSA) has increased in contemporary society, and moderate to severe OSA has been diagnosed in 49.7% men and 23.4% women^1^. The etiologies contributing to OSA include obesity, anatomic obstruction, degenerated nerve innervations in respiratory-related muscles, and aging^2,3^. Untreated OSA leads to higher mortality, higher comorbidities with stroke, cerebral vascular events, hypertension, diabetes mellitus, coronary artery disease, carotid stenosis, and depression. In addition, it affects other aspects, such as roommates’ health^4^.

The gold standard for OSA treatment is continuous positive airway pressure (CPAP)^5^. However, poor compliance problems have resulted in treatment failure. Therefore, the long-term survival of patients with OSA receiving CPAP remains a major concern^6^. Sleep surgery is considered an alternative treatment strategy for patients who cannot tolerate CPAP^7^, and the success rates of uvulopalatopharyngoplasty (UPPP) in moderate and severe OSA patient groups were 42.5% and 26.5%, respectively^8^. Friedman Staging System Stage I had a 80.6% success rate in patients who underwent UPPP^8^. However, the UPPP success rate is unsatisfactory in patients with small tonsils and a bulky tongue base, and additional therapy for the tongue base is recommended, particularly for patients with anatomical tongue-base obstruction during sleep^9^.

Various surgical methods have been adopted for bulky tongue base treatment, including transoral robotic surgery, coblation endoscopic lingual lightening, submucosal minimally invasive lingual excision, tongue base radiofrequency reduction, and tongue base suspension^10,11^. Tongue base suspension is beneficial in patients with OSA who are also diagnosed with tongue base collapse, tongue base atonia, glossoptosis, or an anatomically bulky tongue base^11^. In one study, the surgical success rate during a 3–36-month follow-up period with grade C recommendation was 73.7%^12^. Elsewhere, the retropalatal and retrolingual collapse decreased and spaces increased after tongue base suspension with UPPP^13^. However, whether this combined surgery can improve sleep arousals, sleep quality, rapid eye movement (REM) sleep percentile, and non-REM stage sleep percentile in patients with OSA has been insufficiently discussed. The present study therefore investigated whether tongue base suspension with UPPP can improve sleep quality in patients with OSA diagnosed as having small tonsils and a bulky tongue base.

## Materials and Methods

The charts of 32 male and four female patients with OSA underwent tongue base suspension with UPPP from 2012 through 2015 were reviewed. Retrospectively registered with Research Ethics Committee of China Medical University and Hospital (No. CMUH-102-REC2–005). Surgical success was defined as a >50% AHI reduction rate and having the AHI reduction less than 20 per hour according to Sher’s criteria^14^. The following data, i.e. age, gender, height, weight, and history were collected. All patients underwent upper airway examination with awake nasopharyngoscopy in a clinic and received drug-induced sleep endoscopy (DISE) in an operating room before surgery. The VOTE (velum, oropharyngeal-lateral walls, tongue base, and epiglottis) grading system was adopted to determine the degree and configuration of disease-related obstruction during awake endoscopy and DISE^15^. All of the patients were diagnosed as having small tonsils (size < grade II) and a bulky tongue base (modified Mallampati index > III), with at least 50% anterior–posterior (A–P) velum and tongue base collapse by DISE before operation day. The awake Muller maneuver and sleep endoscopy were used to make the diagnoses. The Institutional Review Board of China Medical University approved this retrospective study, and the patients provided informed consent for their medical record review.

### Surgical Intervention

Tongue base suspension (Repose System, Metronic, USA) with UPPP was performed simultaneously as a single-stage surgery under general anesthesia in all of the patients. Specifically, the patients underwent awake transnasal laryngoscopy and Muller maneuver test, and were confirmed to have A–P velum and tongue base collapse (grade I–II, according to the VOTE staging of sleep endoscopy). In addition, all of the patients underwent DISE (propofol), which further confirmed the presence of tongue base collapse (collapse at the velum and tongue base levels). Polysomnography (PSG) was also used to verify that all of the patients had OSA.

### Tongue Base Suspension

**Figure 1:**
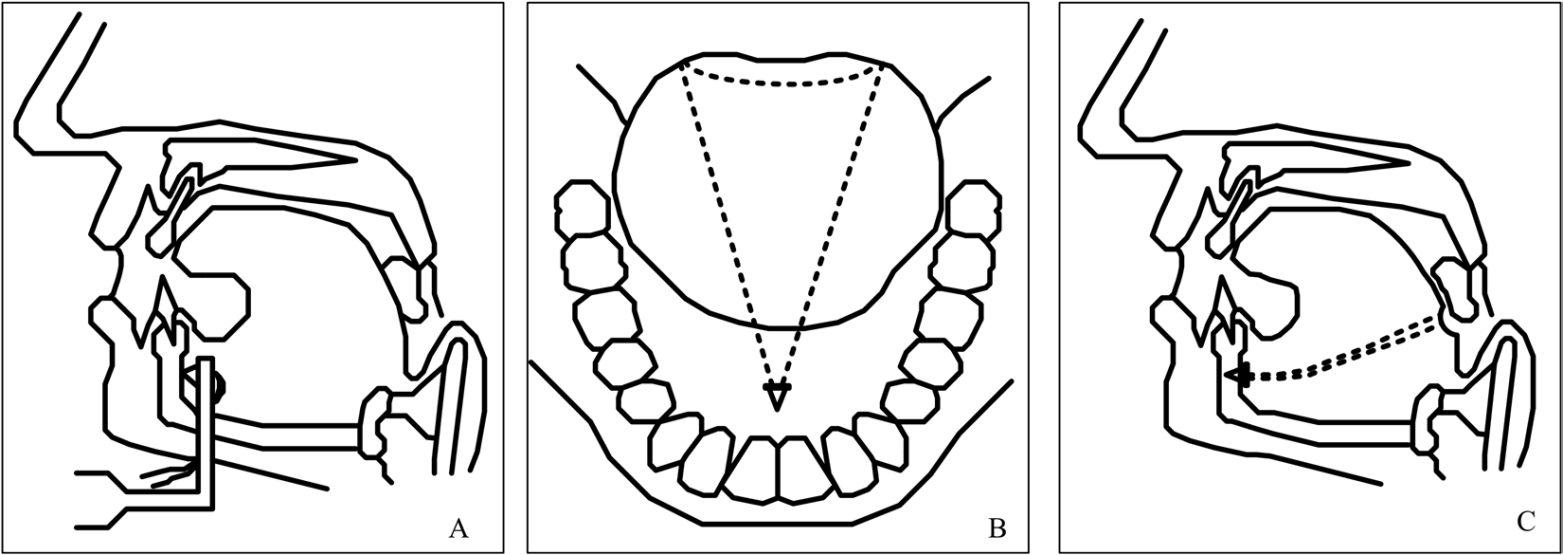
Tongue base suspension.

Tongue base suspension was performed through a 2-cm incision in the submental area. The subcutis was dissected to expose the submental tuberosity, and a titanic screw was nailed to the inner side of the mandible just beneath the tuberosity. One of the two polypropylene sutures attached to the nail was first passed through the left tongue base (Fig. 1A). Subsequently, the needle with a 3–0 vicryl loop was passed through the right tongue base. With the help of a free round needle, the left tongue base polypropylene suture was passed across the right tongue base suture loop (Fig. 1B). The 3–0 vicryl suture loop was then pulled back to the submental area to bring the polypropylene suture close to the submental nail area. Finally, the two polypropylene sutures were tied and fixed at the submental area to obtain maximal tightness (Fig. 1C).

The submental skin area was closed with a 4-0 vicryl suture for the subcutis wound, and with a 5-0 nylone suture for the skin wound with a 5-0 penrose drain fixed at submental wound area. To prevent substantial vessel injury, the needle passer was maintained in the midline tongue position, and in the lateral position while palpating the tongue base to ensure that the great vessels were kept separate from the penetration area at the tongue base. All of the patients received a 2-day course of postoperative antibiotics and steroid and nonsteroid anti-inflammatory drugs at the hospital before discharge.

### Polysomnography

All of the patients underwent full-night PSG at the China Medical University Hospital sleep center under sleep technicians’ supervision in a temperature-controlled and noise-free room. The technicians performed EEG, submental electromyography (EMG), and electro-oculography studies on the patients before they slept. The thermistors detected oral and nasal airflow, and the pulse oximeter recorded patients’ oxygen saturation levels. The technicians and sleep physicians evaluated patient sleep PSG data and scored them based on the apnea–hypopnea index (AHI). The AHI is defined as the summation of total apnea and hypopnea during a patient’s sleep period per hour, and is used determine sleep apnea severity. Apnea was defined as the stop of flow or flow <10% of the average flow for more than 10 seconds with 4% oxygen desaturation. Obstructive hypopnea was defined as >30% decreased flow compared with average baseline breath for more than 10 seconds, also with 4% oxygen desaturation. Notably, the sleep technicians and sleep physicians who provided the scores were blinded to the intervention performed by the surgeon.

### Preoperative and Postoperative Assessment

The preoperative and postoperative sleep quality parameters were evaluated based on arousal indices separately measured as the spontaneous EEG arousal index, which was defined as arousals per sleep hour without explained causes before arousal events and the spontaneous appearance of arousal waves on the EEG for more than 3 seconds. Arousal was defined by the appearance of theta and alpha waves with >16 Hz frequencies and were not revealed in spindles of the EEG study. In addition, at least 10 seconds of sleep should occur before the appearance of arousal, and arousal duration should be more than 3 seconds along with an increase in chin EMG^16^. The respiratory arousal index was defined as respiration events (apnea or hypopnea) occurring immediately before arousal events per hour of sleep. The periodic leg movement (PLM) arousal index was defined as leg movement immediately before arousal events per sleep hour. The total sleep time changes were evaluated based on EEG study, REM sleep percentile, and Epworth sleep scale (ESS) scores. The postoperative respiratory, PLM, and spontaneous arousal indices, slow wave sleep (SWS), sleep efficiency, total sleep time, REM sleep percentile, and ESS scores were compared between the successful surgery and surgical failure groups during a 1-year follow up. Four total arousal parameters and PLM, respiratory, and spontaneous EEG arousal indices were evaluated to assess patients’ curative effect.

### Statistical Analysis

Data are presented as means ± standard deviations. The Wilcoxon test was used to compare the pre- and post-operative results, and the Mann–Whitney test was performed to analyze the differences between the responders and nonresponders. Differences were evaluated using analysis of covariance, with disease severity as a covariable. Data were analyzed using SPSS (version 18; SPSS Inc, Chicago, IL, USA), and P < 0.05 was considered statistically significant.

## Results

This study included 32 male and four female patients with OSA. The average age of all patients was 40.2 years (18–58 years; Table 1). The values of AHI and body mass index (BMI), as well as the ESS scores were within normal ranges before and after surgery (Table 2). The successful surgery and surgical failure groups had 24 (66%)and 12 (34%) patients according to Sher’s criteria, respectively; no significant differences were observed between the two groups in age, AHI score, BMI score, ESS score, or sleep stages before surgery.

**Table 1:** Patient grouped by surgical outcome before surgery.

Values	Success group^a^ (n = 24)	Failure group^b^ (n = 12)	*P*
Age (SD), yrs	38.06(8.91)	42.45(8.13)	0.12
**Gender**			
Male/Female	22/2	10/2	0.56
BMI (SD), kg/m^2^	26.25(2.73)	27.49(3.12)	0.11
REM arousal index (SD)	18.27(23.45)	13.91(19.46)	0.29
Non-REM arousal index (SD)	22.87(23.09)	15.32(20.44)	0.18

BMI, body mass index; REM, rapid eye movement.

^a^AHI > 50% reduction and AHI < 20; ^b^AHI reduction not over 50% or AHI > 20.

**Table 2:** Patients characteristics at baselines.

Values	Total (n = 36)			Success group^a^ (n = 24)			Failure group^b^ (n = 12)		
	Before	After	*P*	Before	After	*P*	Before	After	*P*
Age(SD), yrs	40.22(9.14)			38.08(8.95)			42.42(8.15)		
AHI(SD), times/ hour	25.14(17.53)	17.51(18.92)	0.04*	20.93(19.37)	3.58(3.52)	0.00*	29.37(15.02)	31.63(17.46)	0.33
BMI(SD), kg/m^2^	26.85(2.92)	26.13(2.85)	0.18	26.24(2.77)	25.52(2.37)	0.22	27.46(3.13)	26.84(3.28)	0.28
ESS(SD)	11.86(4.31)	10.20(4.33)	0.05	10.67(4.02)	9.45(4.08)	0.19	13.12(4.41)	10.92(4.59)	0.08
Sleep efficiency(SD),%	79.98(18.45)	81.90(16.54)	0.45	79.05(11.97)	87.41(7.34)	0.03*	81.12(16.70)	74.87(21.93)	0.45
SWS (SD),%	20.67(10.47)	23.76(10.28)	0.06	18.32(9.26)	23.37(7.08)	0.02*	23.59(10.72)	24.26(13.53)	0.60

AHI, apnea–hypopnea index; BMI, body mass index; ESS, Epworth sleep scale; SWS, Slow wave sleep.

^a^reach Sher’s criteria; ^b^not reach to Sher’s criteria. *P < 0.05.

PLM, respiratory, and spontaneous EEG arousal indices were evaluated to assess patients’ curative effect. The SWS, and sleep efficiency are significantly increased in surgical success group (Table 2). In addition, a comparison between the pre- and post-operative arousal parameters in the successful surgery and surgical failure groups revealed that two of the four arousal parameters in the respiratory arousal index decreased considerably but PLM and spontaneous EEG arousal indices did not decrease in the successful surgery group (Tables 3 and 4). No arousal (respiratory, PLM, spontaneous)-related differences were observed in the surgical failure group, and no significant differences were observed in the PLM or spontaneous EEG arousal indices in the successful surgery group. However, the REM sleep percentile significantly increased from 24.7 ± 6.9 to 41.9 ± 5.2 (% total sleep time, TST) in the successful surgery group (P = 0.001). By contrast, the REM sleep percentile decreased from 22.6 ± 8.7 to 19 ± 9.3 (%TST) in the surgical failure group (P = 0.106).

**Table 3:** Parameters of total arousal, PLM-related, respiratory-related, and spontaneous EEG in successful group (n = 24).

Values	Total			Non-REM			REM		
	Before	After	*P*	Before	After	*P*	Before	After	*P*
Total Arousals(SD), times/hour	36.77(9.65)	15.02(8.37)	0.01*	38.23(20.51)	15.87(8.78)	0.01*	27.13(20.88)	12.72(10.86)	0.01*
PLM-related(SD), times/hour	0.36(0.85)	0.66(1.14)	0.19	0.37(0.85)	0.77(1.33)	0.15	0.23(0.97)	0.24(0.59)	0.46
Respiratory-related(SD), times/hour	22.03(12.07)	3.29(5.73)	0.01*	22.88(13.07)	2.65(4.75)	0.01*	18.23(13.49)	5.37(10.31)	0.01*
Spontaneous EEG(SD), times/hour	14.48(13.81)	11.32(5.92)	0.17	15.16(14.93)	12.55(7.17)	0.23	8.89(7.38)	7.22(3.65)	0.18

REM, rapid eye movement; PLM, periodic leg movement; EEG, electroencephalography. *P < 0.05.

**Table 4:** Parameters of total arousal, PLM-related, respiratory-related, and spontaneous EEG in failure group (n = 12).

Values	Total			Non-REM			REM		
	Before	After	*P*	Before	After	*P*	Before	After	*P*
Total Arousals(SD), times/hour	23.57(17.41)	29.25(15.87)	0.18	24.67(18.75)	31.82(17.88)	0.17	16.75(19.24)	18.37(13.41)	0.40
PLM-related(SD), times/hour	0.31(0.75)	0.84(2.31)	0.23	0.43(0.98)	1.12(3.24)	0.22	0.07(0.04)	0.21(0.67)	0.10
Respiratory-related(SD), times/hour	14.87(18.55)	19.84(17.16)	0.24	15.34(20.43)	21.42(19.31)	0.23	13.92(19.47)	23.87(41.93)	0.23
Spontaneous EEG(SD), times/hour	7.92(5.91)	8.68(3.41)	0.36	8.94(6.27)	8.98(3.41)	0.42	2.88(3.16)	10.24(19.37)	0.10

REM, rapid eye movement; PLM, periodic leg movement; EEG, electroencephalography.

## Discussion

Surgical success was defined as a >50% AHI reduction rate and having the AHI reduction less than 20 per hour according to Sher’s criteria. Earlier, surgical success was defined by the respiratory disturbance index, including the AHI and respiratory-related arousal events (RERA), which remain assessment tools for surgical and nonsurgical treatment outcomes^17^. Therefore, surgical treatment is beneficial in reducing respiratory arousal in patients with OSA. However, studies on OSA-related sleep arousal are limited, and surgical outcome assessment tools do not calculate RERA in current practices^18^. In our study, we evaluated the sleep quality changes by assessing the respiratory, spontaneous, and PLM arousal indices. In addition, the REM percentile and ESS score changes were considered to be objective and subjective sleep quality changes, respectively. Overall, this study presented a detailed investigation of sleep quality after tongue base suspension with UPPP as a single modality surgery in patients with OSA who have also been diagnosed with small tonsils and a bulky tongue base.

Tongue base suspension with UPPP has had a 70% (20–88.9%) success rate in patients with OSA, and a long-term success rate of 78% when combined with nasal surgery^12,19,20^. However, the anatomical base selection of patients has been insufficiently explored; only one long-term study on tongue base suspension and pharyngeal surgery with a 2-year follow-up period reported a 52% success rate. This study revealed a 66% success rate in patients with OSA diagnosed as having >50% velum and tongue base collapse. Moreover, although the success rate might decrease with time and the cutting effect or tissue loosening might limit the long-term effects of tongue base suspension surgery, a significantly decreased respiratory arousal index, and increased SWS and sleep efficiency were observed in the successful surgery group. Although only a few studies have addressed sleep quality problems by performing sleep surgery, it cannot be inferred that sleep surgery is not beneficial to improving sleep quality, particularly because successful sleep surgery was found to reduce respiratory arousal events in the present study. We also determined that the REM sleep percentile increased in the successful surgery group. To eliminate the variations due to artificial patient selection, we analyzed the pre- and post-operative SWS and sleep efficiency in the successful surgery and surgical failure groups, and no significant variations were observed between the two groups after surgery (Table 2). However, the successful surgery group showed significantly reduced respiratory arousal index, increased sleep efficiency, and increased SWS even after a 1-year follow up. Tongue-base-suspension-related complications, such as odynophagia, infection, dysphagia, dysarthria, limited tongue motion, mouth floor cyst, mouth floor hematoma, tongue atrophy, and tongue base abscess, have all been reported^21–23^. Here, only a few temporary complications, including temporary dysphagia (3/36), temporary dysarthria (3/36), and one mouth floor hematoma (1/36), were reported, and they subsided after a 3-month follow up. Notably, dysphagia, choking, or cough-related arousals were not reported in any of the studied patients during the 1-year follow-up period.

Sleep quality was assessed based on the patients’ ESS scores as well as their PSG results. Limited studies have explored surgical results to assess sleep quality and related arousals, although some research has investigated the effects of sleep surgery in children with cerebral palsy and revealed that tongue base suspension with adenotonsillectomy reduces the arousal index^24,25^. However, the present study is the first to assess sleep arousals and sleep quality after tongue base suspension with UPPP in adult patients who have small tonsils and a bulky tongue base. In addition, all of our patients were diagnosed as having velum and tongue base A–P obstruction through DISE and received tongue base suspension with UPPP as a single modality treatment strategy. The two patient groups did not differ significantly in their PLM and spontaneous arousal indices, or in their ESS scores. The successful surgical outcomes improved sleep quality by reducing the respiratory arousal index and increasing the REM sleep percentile during the 1-year follow-up period. We suggest that the PLM and spontaneous arousals are not related to obstructive respiratory events and can simply be considered as other neuropsychiatric etiologies in patients with OSA; moreover, these arousals could not be directly reduced by sleep surgery.

Some limitations in the present study must be noted. First, although ST90 (sleep time with oxygen saturation below 90%) was reported as a predictive outcome for tongue base suspension with UPPP^26^, significant predictive outcomes were not analyzed in our study due to small sample size. Studies with a longer follow-up period and larger sample sizes are warranted to confirm the aforementioned findings. Second, a bulky tongue base was diagnosed based on Mallampati score or Friedman tongue position in the clinic and DISE in the operation room, rather than through a detailed assessment based on lingual tonsil enlargement or performing tongue base muscle hypertrophy. Therefore, sleep cine magnetic resonance imaging and sleep computed tomography imaging studies are warranted^27,28^. We argue that successful tongue base suspension with UPPP can reduce sleep-related arousals and ESS scores, as well as increase REM sleep percentiles in patients with OSA. The overall surgical success rate was 66% during our 1-year follow up, and studies on long-term outcomes are ongoing.

## Conclusion

Tongue base suspension with UPPP is a successful surgical treatment method for patients with OSA diagnosed as having Fujita type III collapse. Increased total sleep time, SWS, and sleep efficiency significantly reduced respiratory arousal index were observed in the successful surgery group.
